# Cost-effectiveness of pharmacogenetic-guided treatment: are we there yet?

**DOI:** 10.1038/tpj.2017.21

**Published:** 2017-06-13

**Authors:** M Verbelen, M E Weale, C M Lewis

**Affiliations:** 1 MRC Social, Genetic and Developmental Psychiatry Centre, Institute of Psychiatry, Psychology and Neuroscience, King’s College London, London, UK; 2 Division of Medical and Molecular Genetics, Faculty of Life Sciences and Medicine, King’s College London, London, UK

## Abstract

Pharmacogenetics (PGx) has the potential to personalize pharmaceutical treatments. Many relevant gene–drug associations have been discovered, but PGx-guided treatment needs to be cost-effective as well as clinically beneficial to be incorporated into standard health-care. We reviewed economic evaluations for PGx associations listed in the US Food and Drug Administration (FDA) Table of Pharmacogenomic Biomarkers in Drug Labeling. We determined the proportion of evaluations that found PGx-guided treatment to be cost-effective or dominant over the alternative strategies, and estimated the impact on this proportion of removing the cost of genetic testing. Of the 137 PGx associations in the FDA table, 44 economic evaluations, relating to 10 drugs, were identified. Of these evaluations, 57% drew conclusions in favour of PGx testing, of which 30% were cost-effective and 27% were dominant (cost-saving). If genetic information was freely available, 75% of economic evaluations would support PGx-guided treatment, of which 25% would be cost-effective and 50% would be dominant. Thus, PGx-guided treatment can be a cost-effective and even a cost-saving strategy. Having genetic information readily available in the clinical health record is a realistic future prospect, and would make more genetic tests economically worthwhile.

## Introduction

Pharmacogenetics (PGx) studies the relationship between genetic variation and inter-individual variability in drug response in terms of efficacy and safety. Hence, PGx knowledge can be used to tailor pharmaceutical treatment to the genetic make-up of the patient. Several robust, well-replicated PGx associations exist, for example, the association of HLA-B*5701 with abacavir hypersensitivity, HLA-B*1502 with carbamazepine-induced Stevens–Johnson syndrome/toxic epidermal necrolysis, and *VKORC1* and *CYP2C9* with warfarin dosing.^1, 2, 3^ Accordingly, the US Food and Drug Administration (FDA) includes information about PGx associations in many drug labels in a wide range of therapeutic areas.^4^ These PGx drug labels cover tests that are commonly used, but also include weaker genetic associations that are reported without requiring adjustments to pharmaceutical treatment. Most drugs with mandatory genetic testing are used in oncology, but PGx tests in other therapeutic areas are already being offered by laboratories and some have become part of standard clinical practice.^5, 6^

Personalizing drug treatments through PGx testing could improve their efficacy and safety, as well as reduce costs.^7^ However, as health-care resources are finite, it is important that the cost-effectiveness of novel PGx-guided treatment strategies is assessed in addition to their clinical utility before they are widely applied. Economic evaluations, which compare costs and outcomes of at least two competing interventions, are a useful tool to inform decision making and prioritize health-care spending. In the context of PGx testing, a pharmaco-economic study might contrast PGx-guided treatment with standard treatment (ST) with the same drug, or with an alternative drug that does not require genetic testing, or with both alternatives. When the PGx strategy is found to be more effective at an acceptable additional cost (cost-effective) or more effective at a lower cost (cost-saving or dominant), this provides a strong argument for the implementation of PGx testing.

Previously published literature reviews of PGx-guided treatment and personalized medicine reported that the majority of PGx strategies were cost-effective or even dominant, though they noted that there was large heterogeneity in methodology between studies.^8, 9, 10, 11, 12^ Concerns over the quality of the early economic evaluations of PGx-guided treatment have been raised, but the quality is generally considered to have improved over time.^13, 14, 15, 16^

Our review of pharmaco-economic studies of PGx-guided treatment provides an update on the literature in this rapidly evolving field (the most recent previous review covered studies up to early 2013 (ref. 10)). Furthermore, we include a more extensive range of economic evaluations, whereas recent literature reviews were limited to cost utility analyses (CUAs) only.^10, 11^ We also assessed the impact of freely available genetic information on the cost-effectiveness of PGx-guided treatment. We adopted a narrow definition of PGx, limiting our scope to consideration of variation in germline DNA. In contrast to tests on tumour, viral or bacterial DNA, germline DNA has the advantage that genetic variants need to be typed only once, and results remain relevant throughout a patient’s life.

## Materials and methods

### Data sources and search strategy

The FDA Table of Pharmacogenomic Biomarkers in Drug Labeling lists FDA-approved drugs that include PGx information on their drug label along with the biomarker gene (accessed on 18 September 2015).^4^ We used this table to identify drugs for which there is a genetic variant associated with the drug efficacy, safety or dosing. We excluded non-germline genetic biomarkers, for example, mutations in viral or tumour DNA.

We then searched for the selected drugs and biomarkers in the National Health Service Economic Evaluations Database (NHS EED), a UK Department of Health and National Institute for Health Research-funded registry of economic evaluations of health and social care interventions.^17, 18^ This resource includes CUAs, cost-effectiveness analyses (CEAs), cost–benefit analyses (CBAs—see below for definitions of these terms) and commentaries by the Centre for Reviews and Dissemination of the University of York. Funding of the NHS EED ceased in March 2015 and the latest database update was December 2014.

For each drug included in our study, the NHS EED was searched for economic evaluations that contain (1) the drug name and (2) the specific gene from the FDA label or the search terms genetic, genotype, genotypic, pharmacogenetic or pharmacogenomic in any field. We only included studies that compared a PGx-guided treatment strategy with at least one alternative strategy.

We also searched PubMed to identify more recent papers (until September 2015) and any other studies missed by the NHS EED search. We searched for articles that included (1) the name of the drug and (2) the specific gene mentioned in the FDA label or the search terms genetic, genotype, genotypic, pharmacogenetic or pharmacogenomic in the title or abstract, and (3) Cost-Benefit Analysis as a Medical Subject Headings term. In addition, the reference lists of retrieved publications were used to identify additional studies missed in our database searches.

### Overview of economic evaluation methodology

Measuring and comparing costs and health outcomes is essential in a pharmaco-economic study. Whereas costs are naturally expressed in monetary units, the effect of a healthcare intervention can be expressed in different ways. In CUAs, health outcomes are assessed as quality-adjusted life years (QALYs), which measure the expected number of post-treatment years of life accounting for the quality of life. QALYs allow comparisons of treatment strategies across therapeutic areas and populations, but are an abstract concept (‘quality’ is hard to define) and their validity has been questioned.^19^ CEAs evaluate the effect of an intervention in terms of a disease or treatment specific measure, for example the number of adverse events avoided, the change in score on a depression rating scale or time taken to remission. CBAs quantify treatment outcome in purely monetary terms.

Furthermore, the perspective of a pharmaco-economic study determines which costs and benefits are taken into account. These can be limited to costs to the public health-care system or private insurers, for example, staff salaries, drugs and equipment costs, or may include broader costs such as productivity losses and informal care. Commonly used perspectives are the third-party payer and societal perspective, but some studies take a hospital or patient perspective.

The incremental cost-effectiveness ratio (ICER) summarizes the difference in costs and health outcomes between a PGx-guided strategy and ST:


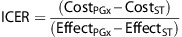


**Figure 1:**
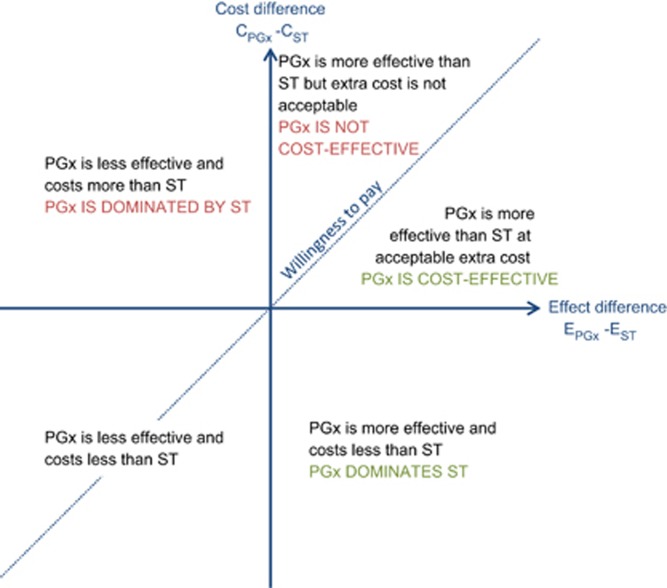
Cost-effectiveness plane of pharmaco-economic studies. PGx, pharmacogenetics-guided treatment; ST, standard treatment.

If the PGx treatment reduces costs and achieves a better outcome than the ST, then the PGx strategy dominates the ST. Contrarily, if the PGx option costs more but is less effective than the ST, then the PGx treatment is dominated by the ST (Figure 1). When one treatment comes at a higher cost but is also more effective than the other, the ICER is compared to a willingness-to-pay threshold to determine cost-effectiveness. Generally, ICERs up to £20 000–£30 000 per QALY (or $30 000–$50 000 per QALY) are considered cost-effective.^20^ As costs, health outcomes and willingness-to-pay thresholds differ between countries, or may differ according to the assumptions and perspectives adopted, economic studies evaluating the same PGx test may come to different conclusions.

### Analyses

We extracted key parameters from the reviewed economic evaluations, including the unit of outcome, country, perspective, ICER if applicable and the conclusion regarding the cost-effectiveness of the PGx testing strategy (the interpretation of the result as described in the publication). A parameter of particular interest is the cost of the genetic test, as this can significantly affect the cost-effectiveness of the PGx testing strategy and may change over time. To allow comparison between studies, the price of the genetic test was corrected for inflation and converted to US dollars estimated at 2014 levels (2014 US$).

A stepwise linear regression model was fitted to test whether publication year, geographic region (Asia, Oceania, United States and Canada or EU) or perspective (health care, society or other) had an influence on the price of genetic testing. A stepwise logistic regression model was also used to investigate whether publication year, geographic region, perspective, cost of genetic test, genetic variant (*HLA*, *TPMT* or other) or outcome (QALY or other) was associated with the PGx testing strategy being cost-effective. Statistical analysis was performed in R (version 3.1.2, R Foundation for Statistical Computing, Vienna, Austria).

We estimated the impact of freely available genetic information on the conclusions regarding the cost-effectiveness of PGx-informed strategies. The ICER under assumption of free genetic testing was calculated by adjusting the cost of the PGx-guided treatment for the cost of the test as reported in the reviewed studies


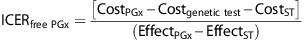


When insufficient details were provided to estimate the ICER_free PGx_, it was assumed that free genetic testing could not worsen the conclusion regarding PGx-guided treatment. For example, when a study found the PGx strategy to be cost-effective, we assumed that PGx-guided treatment with free genetic testing would also be at least as cost-effective.

## Results

### Description of studies

**Figure 2:**
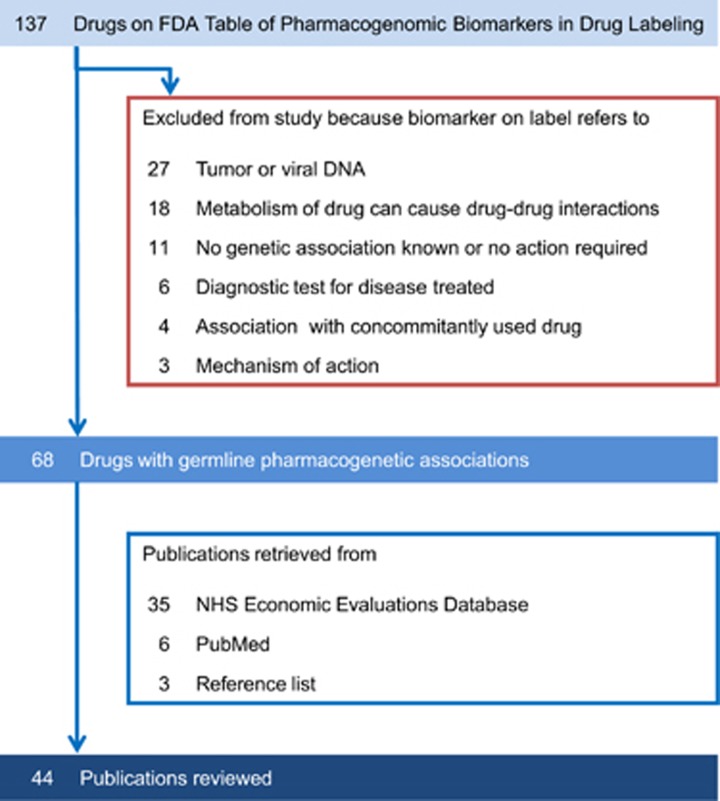
Number of drugs and publications included in literature review.

The FDA Table of Pharmacogenomic Biomarkers in Drug Labeling listed 137 distinct drugs, of which 68 met our inclusion criteria (Figure 2). These drugs were from diverse clinical specialties, including cancer (11 drugs), infectious diseases (10 drugs), psychiatry (9 drugs) and neurology (8 drugs) (Table 1). Our literature search yielded economic evaluations for only 10 of these 68 drugs (14.7% Table 2). All publications related to a single drug, except for one study investigating a PGx testing strategy for carbamazepine and phenytoin treatment, which assumed both drugs to be interchangeable in terms of costs, efficacy and safety.^21^ To avoid duplication of studies in our review, this publication was counted as a carbamazepine study (there were no other publications on phenytoin).

We retrieved 44 economic evaluations that investigated the cost-effectiveness of a PGx-informed strategy (Table 2). Full details of the reviewed studies and extracted information are given in Supplementary Table 1. The earliest study included was published in 2000 and over 70% of studies were published in 2009 or later. Most publications were CUAs (30 studies, 68%) or CEAs (12 studies, 27%), with only two CBAs (5%). A health-care system perspective was adopted in 18 studies (41%), a societal perspective in 10 papers (23%), a third-party payer perspective in 5 studies (11%) and 11 papers (25%) did not state a clear perspective. In all, 20 studies (45%) were conducted in North America, 11 (25%) in Europe, 6 (14%) in Asia and 3 (7%) in Oceania; 4 studies (9%) did not specify a country. Warfarin had the most economic evaluations (12 studies), followed by azathioprine (9 studies); clozapine and mercaptopurine had only 1 economic evaluation each (Table 2).

### Cost-effectiveness of PGx-informed treatment

**Figure 3:**
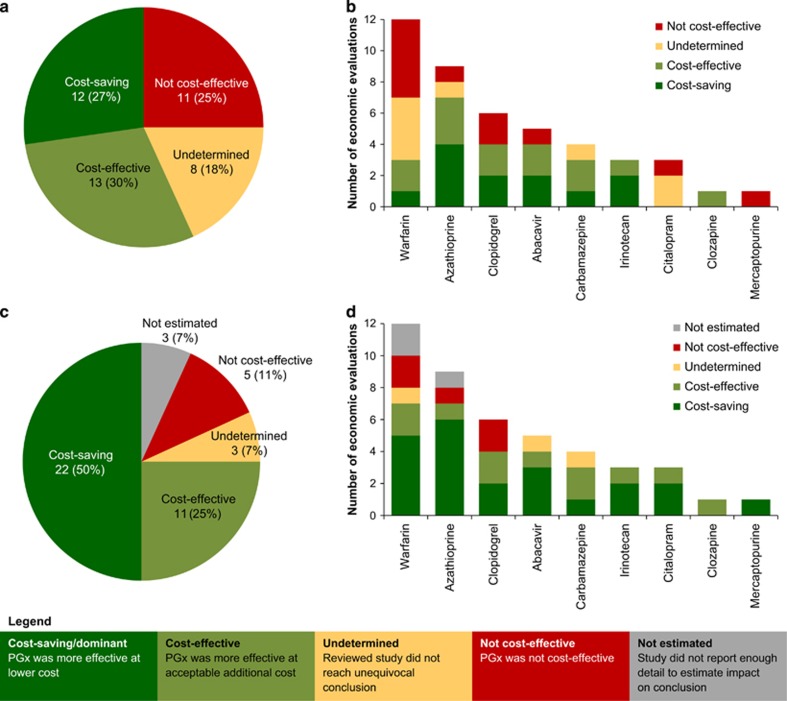
Conclusions of reviewed economic evaluations regarding cost-effectiveness of PGx testing strategy (**a**) overall and (**b**) by drug, and estimated conclusions in scenario of no extra cost for genetic information (**c**) overall and (**d**) by drug. PGx, pharmacogenetics-guided treatment.

We assessed the overall conclusions regarding cost-effectiveness of each PGx study. Over half of the 44 economic evaluations took a favourable view of the PGx-guided strategy: in 12 studies (27%) it was dominant (cost-saving) and in 13 studies (30%) it was cost-effective. Eleven publications (25%) found PGx testing not cost-effective and 8 studies (18%) did not reach a definitive conclusion (Figure 3a). The majority of economic evaluations concluded in favour of PGx testing for azathioprine (7 out of 9 studies), clopidogrel (4 out of 6 studies), abacavir (4 out of 5 studies), carbamazepine (3 out of 4 studies), irinotecan (3 out of 3 studies) and clozapine (1 study) (Figure 3b). Although warfarin had the highest number of economic studies, they reached diverging conclusions: 3 studies found PGx-guided dosing cost-effective, 4 studies were inconclusive and 5 studies concluded it was not cost-effective. No studies found unequivocally that PGx-guided citalopram (3 studies) or mercaptopurine (1 study) treatment was cost-effective.

We assessed the effect of study characteristics on the probability of concluding in favour of the PGx strategy. A logistic regression model detected that CUAs (studies using QALYs as outcome measure) were less likely than CEAs and CBAs to find the genetic testing strategy cost-effective (odds ratio=0.13, *P*-value <0.05). However, there is no clear explanation for this and it may be a spurious result due to the relatively small sample size of 44 economic evaluations.

### Effect of cost of genetic test on cost-effectiveness of PGx-informed treatment

**Figure 4:**
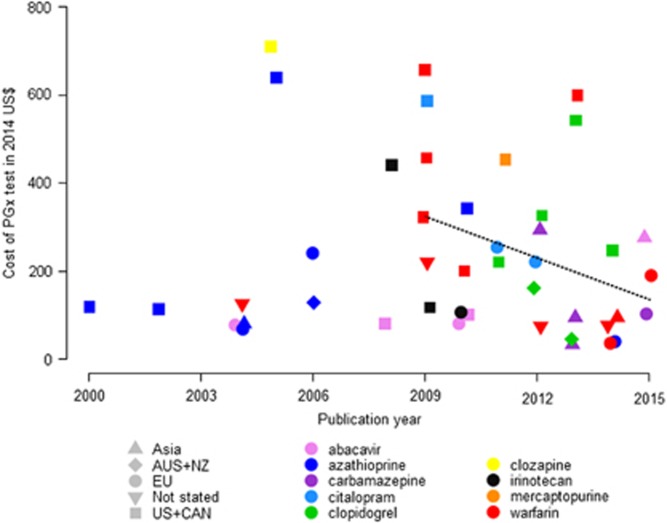
Cost of pharmacogenomics (PGx) test as reported in the reviewed economic evaluations over time, with fitted regression since 2009 (dotted line).

The cost of genetic testing is an important parameter of economic evaluations of PGx interventions. After correcting for inflation and converting to 2014 US$, the cost of genetic testing quoted by the reviewed studies ranged between US$33 and US$710 with a median value of US$175. The price of genetic tests decreased slightly over time (not statistically significant) and this trend was more pronounced since 2009, the period when most economic evaluations were published (*P*-value <0.05; Figure 4). Prices were on average higher in the United States and Canada than other regions of the world (mean United States and Canada: US$363.65; mean other regions: US$131.80; *P*-value <0.05). We noted a wide variability in prices of tests for the same drug. For example, the lowest price quoted for warfarin PGx testing was US$36 in a 2014 UK-based study,^22^ while US$600 and US$657 were used in a 2013 Canadian and 2009 US study, respectively.^23, 24^ The prices for clopidogrel PGx testing also varied considerably: from US$45 (2013 Australian study) to US$ 543 (2013 US study).^25, 26^

Given the decreasing costs of genetic testing and its increasing availability, we looked ahead to a possible future where genotype information might be readily available, at negligible cost, for all patients as part of their electronic health record. Thirty-three economic evaluations (75%) would support PGx-guided treatment under this scenario, with 11 studies (25%) finding it cost-effective and 22 studies (50%) considering it dominant and cost-saving (Figure 3c). Five studies (11%) would still conclude that PGx testing was not cost-effective, while 3 studies (7%) would be inconclusive. A separate set of 3 studies had to be excluded, because the impact of free genetic testing could not be estimated. We note that the effect of freely available genetic information can be striking for some drugs. None of the published studies for citalopram and mercaptopurine found PGx-informed treatment to be cost-effective, but all studies switched in favour of PGx testing under the negligible test cost scenario (Figure 3d). For the 12 economic evaluations of warfarin, the number of cost-effective studies would increase from 3 to 7 with freely available genetic testing.

## Discussion

We have assessed published economic evaluations comparing the cost-effectiveness of PGx-guided treatment to ST for drugs listed in the FDA Table of Pharmacogenomic Biomarkers in Drug Labeling. The economic evaluations were drawn from the NHS EED database, which includes economic evaluations up to 31 December 2014. An alternative source of economic studies would be the Cost-Effectiveness Registry (CEA Registry) maintained by the Tufts Medical Centre. We opted to use the more comprehensive NHS EED as the CEA Registry is limited to CUAs (measuring health outcomes in QALYs), which would have reduced the number of evaluations available for assessment. Moreover, the CEA Registry was not updated beyond 2014 and it only provides advanced database searches for subscribers and contributors.^18^ A third resource, the Health Economic Evaluations Database curated by John Wiley & Sons, was discontinued in 2014.^27^ As economic evaluations provide evidence for the introduction of PGx testing into clinical practice, we argue that an up-to-date, accessible database would be an important and valuable resource for both health-economic and PGx research.

Few of the FDA-listed drugs have been the subject of published economic evaluations assessing the economic aspects of PGx testing. This was previously also noted by Phillips *et al.*,^11^ who found that only 13% of drugs on the FDA table and only 27% of available genetic tests were accompanied by economic studies. However, it is increasingly the case that clinical utility alone is not sufficient to recommend application of a PGx test in clinical practice, and a favourable economic assessment is therefore of increasing importance. We call for more pharmaco-economic studies in this field, which should be regularly updated to respond to the changing landscape of health-care and, in particular, genetic testing costs.

There are various limitations of our study that need to be taken into account. One is that the economic evaluations reviewed may not be representative of all PGx tests. For example, the economic aspects of PGx-guided treatment are of less relevance in cases where testing is clearly necessary, for example, because it prevents life-threatening adverse events, and economic studies in such cases therefore tend to be lacking. Another possibility is that economic studies focus on PGx tests that are already applied in clinical practice and for which there is an apparent interest. Studies that find genetic testing to be not cost-effective may also be less likely to be published.

Notwithstanding the above issues, economic evaluations also have certain intrinsic limitations. One is that certain inputs into the model are difficult to quantify accurately. For example, parameters such as the response rate, the probability of adverse drug reactions and the cost of managing adverse drug reactions must sometimes be estimated from sparse information. Randomized clinical trials are the preferred source for these input data, but these are not always available. Ideally, the uncertainty in the input estimates should be accounted for in the economic modelling, and sensitivity analyses should be performed to verify how robust the result is to deviations in the inputs, but the level of uncertainty to apply can itself be a matter of subjective opinion, and vary from study to study.

Another intrinsic issue is that context and perspective may influence the conclusion of a study. For example, comparing treatments from the perspective of an insurance company over 5 years will count costs and outcomes differently from looking at the same treatment options from a broader societal perspective. Likewise, economic evaluations are typically country specific, as this determines parameters of costs, treatment options, and rates of non-response and adverse drug reactions. Studies are also time-specific, as their conclusions may become outdated through changes in price, in management of adverse drug reactions or through the availability of new drugs.

In the context of PGx testing, the type of test applied may differ over time and between countries, and this may influence the study result. For example, a lab-developed test is likely to be less expensive than a PGx test which has undergone regulatory approval. Likewise, a multi-variant test may be less expensive than a series of single tests. For example, the PGx GeneSight test uses 44 genetic variants to guide selection of antidepressants for major depressive disorder, with some evidence that the genetic test resulted in higher response rates and was cost-saving.^28, 29^ Indeed, another shift in perspective may occur when PGx information is available for multiple drugs used to treat a specific condition; cost-effectiveness studies will then move from assessing a single drug to evaluating cost-effectiveness at the disease level.

Taken together, these issues imply that cost-effectiveness analyses on their own cannot answer the question of whether or not a certain strategy should be used and funded, but should be considered in conjunction with other factors such as the available resources, the number of patients who benefit from the intervention and other ethical considerations.

Warfarin provides a useful illustration of some of these issues. PGx-guided warfarin dosing was favoured by a US cost-effectiveness study but not supported by a UK study. The UK study compared warfarin (with and without PGx testing), rivaroxaban, apixaban and dabigatran, with costs and health outcomes included from the National Health Service’s perspective.^22^ The US study contrasted warfarin treatment without PGx testing with a strategy where all patients are tested and either receive PGx-guided doses of warfarin or an alternative drug if they have low or high warfarin sensitivity.^30^ The latter analysis took the perspective of the US health-care payers. Both studies estimated costs and benefits on a lifetime horizon, measured health outcomes primarily in terms of QALYs and used *CYP2C9* and *VKORC1* for PGx testing. The UK study concluded that PGx-guided warfarin was cost-effective compared to warfarin with clinically guided dosing, but recommended the use of apixaban, which does not require PGx testing, as the most cost-effective treatment option. In contrast, the US study found that PGx-guided warfarin was cost-effective compared to clinically dosed warfarin and supported the use of PGx testing for warfarin dosing. The US study did not include apixaban or other comparator drugs, which may have influenced the conclusion reached, but many other factors differed between the studies. For example, although the price of the genetic test was twice as high in the US study, this was outweighed by differences in lifetime costs for warfarin in the United States and the United Kingdom. This example highlights the variable factors involved in performing cost-effectiveness analyses, interpreting their results and comparing such studies.

The PGx dosing algorithm for warfarin is often presented as the poster child for the achievements of PGx, because the drug is widely prescribed and implementation of this single-nucleotide polymorphism-based test could have a major impact on health care. However, only one-quarter of studies considered genetic-guided dosing for warfarin to be cost-effective, and the clinical advantage of genetic-guided dosing over standard dosing appears to be small or even non-existent.^31^ Although freely available genetic testing would improve the cost-effectiveness of genotype-guided warfarin dosing, other drugs such as abacavir, where genetic testing for HLA-B*5701 is required by the FDA, might make more convincing PGx success stories.^32^

Our study assessed the characteristics of tests in the reviewed evaluations. We noted that quoted prices for genetic tests in the United States and Canada were higher than that in other countries, although there was also a large between-study variability within these countries. However, higher prices for genetic testing in the United States and Canada did not lead to fewer conclusions in favour of PGx testing, as country was not associated with study outcome. In addition, neither the drug nor the study perspective was significantly associated with the price of testing. Genetic test costs may depend on the method used to determine genetic variants (for example, PCR or measuring enzyme activity), but the reviewed studies did not provide sufficient detail to investigate the impact of this parameter on price. A downward trend in prices for genetic testing is apparent in recent years, and this may continue as new genetic technologies become more accessible and lead to further price reductions.

We show in this study that the cost of genetic testing is an important factor in determining the cost-effectiveness of a PGx-guided treatment strategy. If there was no cost attached to genetic testing, the number of economic evaluations that found the PGx strategy cost-effective increased greatly, such that half of the reviewed studies considered it dominant over the alternative and 75% considered it cost-effective. Freely available genetic testing might be achievable in future as genomic prices fall and the perceived or actual value of genetic information increases. Once genetic tests become a mainstream clinical service, economies of scale will decrease the price of testing still further. For example, the direct to consumer testing company 23andMe offers a genome-wide genotyping service for £149 (United Kingdom, January 2017 price), which includes single-nucleotide polymorphism-based testing for 5 of the 10 drugs covered in this review.^33, 34^ Similarly, the cost of whole-genome sequencing has fallen every year and is now nearing US$1000.^35^ Having genetic information in the electronic health record would allow PGx information to be queried for any new prescription or dosage review. A genetic test would need to be performed only once and this information, safely secured and immediately accessible, could guide treatment throughout the patient’s life.

Even so, PGx-guided treatment will not be cost-effective in all situations. Even under the favourable assumption of freely available genetic testing, it could still be more expensive than the alternative strategy. This sounds counter-intuitive, but genetic testing costs may only be a small part of the costs attached to PGx-informed treatment. Increased costs may arise where the alternative drug for test-positive patients is more expensive, and this is exacerbated whether the test has a high proportion of false-positive results. For example, patients with heart disease or stroke who are *CYP2C19* poor metabolizers may be prescribed the more expensive ticagrelor in place of clopidogrel (which is metabolized into its active form by *CYP2C19*).^25^ Thus, even if genetic information is freely accessible, economic evaluations of PGx testing are still relevant and necessary.

The economic evaluation studies reviewed here show that PGx has a positive impact on health-care quality and costs. Over half of reviewed studies concluded that the PGx-informed treatment strategy is more cost-effective than the alternatives considered under present-day economics. Only one in four economic evaluations found the genetic testing option unequivocally not cost-effective. This encouraging finding, with an even bigger projected benefit under low-cost genetic typing, suggests that PGx testing has the potential to be a cost-effective or even cost-saving intervention. It therefore seems likely that PGx testing will become a core clinical service, particularly as projects such as the 100 000 Genomes Project pushes genomics to become part of health-care infrastructure and as electronic health records become increasingly effective.^36^

## Figures and Tables

**Table 1:** Drugs from the FDA Table of Pharmacogenomic Biomarkers in Drug Labeling included in literature review

*Therapeutic area*	*Count*	*Drugs*
Oncology	11	Capecitabine, cisplatin, dabrafenib, fluorouracil, **irinotecan**, lapatinib, **mercaptopurine**, nilotinib, pazopanib, rasburicase, thioguanine
Infectious diseases	10	**Abacavir**, chloroquine, dapsone, mafenide, nalidixic acid, nitrofurantoin, primaquine, quinine sulphate, rifampin+isoniazid+pyrazinamide^a^, sulfamethoxazole+trimethoprim^a^
Psychiatry	9	Aripiprazole, atomoxetine, **citalopram**, **clozapine**, fluvoxamine, iloperidone, perphenazine, pimozide, thioridazine
Neurology	8	**Carbamazepine**, clobazam, dextromethorphan+quinidine^a^, divalproex, **phenytoin**, tetrabenazine, valproic acid, vortioxetine
Cardiology	5	Carvedilol, **clopidogrel**, isosorbide+hydralazine^a^, metoprolol, propafenone
Gastroenterology	5	Dexlansoprazole, esomeprazole, metoclopramide, PEG-3350+sodium sulphate+sodium chloride+potassium chloride+sodium ascorbate+ascorbic acid^a^, rabeprazole
Rheumatology	5	**Azathioprine**, carisoprodol, celecoxib, flurbiprofen, pegloticase
Endocrinology	4	Chlorpropamide, glimepiride, glipizide, glyburide
Haematology	3	Eltrombopag, methylene blue, **warfarin**
Analgesic	1	Tramadol
Anaesthesiology	1	Codeine
Dental	1	Cevimeline
Genitourinary	1	Tolterodine
Inborn errors of metabolism	1	Eliglustat
Pulmonary	1	Ivacaftor
Toxicology	1	Sodium nitrite
Transplantation	1	Mycophenolic acid

Abbreviation: FDA, Food and Drug Administration.

Drugs in bold had economic evaluations available.

a Multiple drugs on a single FDA label.

**Table 2:** Drugs for which a PGx-guided strategy was studied in economic evaluation(s)

*Drug*	*Therapeutic area*	*Gene*	*Notes (based on PharmGKB.org*^37^)	*Number of reviewed publications*
Abacavir	HIV	*HLA-B*	Abacavir is contraindicated for HLA-B*5701 carriers as they are at high risk of hypersensitivity reaction.	5 (refs 37, 38, 39, 40, 41, 42)
Azathioprine	Rheumatology	*TPMT*	Carriers of one nonfunctional *TPMT* allele may require reduced azathioprine dose. Carriers of two nonfunctional *TPMT* alleles are at high risk of myelotoxicity and alternative treatment should be considered.	9 (refs 43, 44, 45, 46, 47, 48, 49, 50, 51)
Carbamazepine	Neurology	*HLA-B*, *HLA-A*	Carbamazepine is contraindicated for HLA-B*1502 carriers as they are at high risk Stevens–Johnson syndrome/toxic epidermal necrolysis. HLA-A*3101 has also been associated with hypersensitivity reactions.	4 (refs 21, 52, 53, 54)
Citalopram	Psychiatry	*CYP2C19*, *5-HTTLPR* a, *HTR2A* a	*CYP2C19* poor metabolizers require reduced citalopram starting dose. Polymorphisms in 5-HTTLPR and HTR2A are associated with citalopram response.^55, 56^	3 (refs 57, 58, 59)
Clopidogrel	Cardiology	*CYP2C19*	*CYP2C19* poor metabolizers have reduced response to clopidogrel and alternative treatment should be considered.	6 (refs 25, 26, 60, 61, 62, 63)
Clozapine	Psychiatry	*CYP2D6*, *H2* a*, 5-HTT* a, *5-HT*_*2A*_^a^, *5-HT*_*2C*_^a^	*CYP2D6* poor metabolizers may require reduced clozapine dose. Six polymorphisms in H2, 5-HTT, 5-HT2A and 5-HT2C are associated with clozapine response.^64^	1 (ref. 65)
Irinotecan	Oncology	*UGT1A1*	Patients homozygous for the UGT1A1*28 allele are at higher risk of neutropenia and should receive a reduced starting dose of irinotecan.	3 (refs 66, 67, 68)
Mercaptopurine	Oncology	*TPMT*	Carriers of one nonfunctional *TPMT* allele may require reduced mercaptopurine dose. Carriers of two nonfunctional *TPMT* alleles are at high risk of myelotoxicity and alternative treatment should be considered.	1 (ref. 69)
Warfarin	Cardiology	*CYP2C9*, *VKORC1*	Genetic variation in *VKORC1* and *CYP2C9* explain 40% variance in warfarin dose. Genetic and clinical information can be used to determine starting dose.	12 (refs 22, 23, 24, 30, 70, 71, 72, 73, 74, 75, 76, 77)

Abbreviation: PGx, pharmacogenetics.

a Gene not mentioned on FDA drug label but appears in economic evaluations.
